# Proximity morality in medical school – medical students forming physician morality "on the job": Grounded theory analysis of a student survey

**DOI:** 10.1186/1472-6920-7-27

**Published:** 2007-08-06

**Authors:** Hans O Thulesius, Karl Sallin, Niels Lynoe, Rurik Löfmark

**Affiliations:** 1 Department of Clinical Sciences Malmö, Division of Family Medicine, Lund University, Sweden; 2 Centre for Bioethics, LIME, Karolinska Institutet, Stockholm, Sweden; 3 Research and Development Centre, Kronoberg County Council, Box 1223, SE-351 12 Växjö, Sweden

## Abstract

**Background:**

The value of ethics education have been questioned. Therefore we did a student survey on attitudes about the teaching of ethics in Swedish medical schools.

**Methods:**

Questionnaire survey on attitudes to ethics education with 409 Swedish medical students participating. We analyzed > 8000 words of open-ended responses and multiple-choice questions using classic grounded theory procedures.

**Results:**

In this paper we suggest that medical students take a proximity morality stance towards their ethics education meaning that they want to form physician morality "on the job". This involves comprehensive ethics courses in which quality lectures provide "ethics grammar" and together with attitude exercises and vignette reflections nurture tutored group discussions. Goals of forming physician morality are to develop a professional identity, handling diversity of religious and existential worldviews, training students described as ethically naive, processing difficult clinical experiences, and desisting negative role modeling from physicians in clinical or teaching situations, some engaging in "ethics suppression" by controlling sensitive topic discussions and serving students politically correct attitudes.

**Conclusion:**

We found that medical students have a proximity morality attitude towards ethics education. Rather than being taught ethics they want to form their own physician morality through tutored group discussions in comprehensive ethics courses.

## Background

Medical ethics education differs from other subjects and the importance of formal courses in ethics has been questioned [1]. Some medical schools combine instruction in bioethical principles with teaching of humanities programs [2]. The teaching of ethics varies in Swedish medical schools from interspersed lectures to formal ethics courses. Lately, teachers are developing a common national curriculum in the view of a new Swedish university regulation in 2007 to align with European higher education in the Bologna Process [3]. One of three main outcomes of medical education according to the new regulation is ability to understand and assess values and attitudes. Thus, there is a change process underway regarding medical ethics education in Sweden.

We designed a questionnaire survey in order to elucidate how medical students view the ethics education in medical schools [4,5]. Many students gave input on the ethics course curriculum: Should ethics be taught in lectures or learned through group discussions? Should the ethics course be a separate course among others, or should it be part of other courses with lectures and group discussions interspersed? Should it come early or late in the medical school curriculum? Should the literature be specific ethics literature or novels and short stories with relevant ethical content? From multiple-choice responses we found that strong ethics interest was associated with frequent experiences of physician teachers as good role-models and an absence of poor role models [4]. In the present study we wanted to explore what was going on in medical schools regarding the medical ethics education by analyzing open-ended survey responses together with response data from multiple-choice items.

## Methods

We did a survey on attitudes towards the medical ethics education during 2005 as a request from the delegation of medical ethics of the Swedish Society of Medicine. Swedish medical students from the 1^st^, 5^th ^and 11^th ^(last) term participated. The survey consisted of 14 items of which 10 had a total of 59 multiple-choice response options and generous space for open-ended comments, and 4 items were open-ended only, see Table 1.

**Table 1:** The survey items and numbers of multiple-choice options and open-ended response word count.

Survey items 1–14	Number of multiple choice items	Number of open-ended responses*	Open-ended responses, word count
1. The general outline of the ethics education was valuable	5	36	251
2. The following modes of education were valuable	6	13	76
3. The education was valuable within the following specific fields	18	34	468
4. The education was valuable within the following general fields	7	22	252
5. Which specific or general areas were valuable? Please give examples!	0	55	410
6. This is my general attitude to ethics education	3	66	960
7. Have you experienced the following (regarding physicians/teachers)	4	24	238
8. Have you encountered (good and/or poor role models/situations that affected you)?	2	27	289
9. The following forms of examination were valuable	8	15	108
10. What was your required course literature (in the ethics education)?	0	94	560
11. How important was medical ethics education for you?	6	46	685
12. Please offer suggestions for changes of the design of the ethics course that would improve it	0	110	1352
13. Should ethics education continue during intership and residency? If yes, then how?	2	156	1422
14. Please supply further comments to the questions above.	0	38	1135
TOTAL	**59**	**736**	**8206**

*Total number of responders to open-ended items: 220

### Sample

The overall response rate to the questionnaire survey was 36%, and varied between different centers from 13% to 83%, with a total of 409 respondents, 308 women (75%) and 101 men (25%). More than half (220/409) of the respondents gave one or more written open comments amounting to > 8000 words. These comments were transcribed into Word from handwritten text.

At some centers a whole term would drop out since the responsible teacher failed to hand out the survey. Yet, the response patterns of the different questionnaire items did not differ significantly between schools with low and high response rates when different logistic regression models were applied to the data [4,5]. The most comprehensive open responses came from last term students. Thus the most experienced students gave the biggest input to the analysis of the qualitative data – the main data source for this study.

### Analysis

We analyzed open-ended comments and multiple-choice responses by classic grounded theory (GT) procedures [6-12]. Since classic GT is rarely used and differs from other methods analyzing qualitative and quantitative data we describe and discuss GT a lot in this paper. The GT dictum "all is data" [8] means that we compared both qualitative responses and quantified multiple-choice items in the same analysis. Multiple-choice results were dichotomized, analyzed in logistic regression models, and compared with open-ended responses. GT analysis begins with open coding of data line by line. Codes answer the questions "what is going on?" and "what concept does this data represent" or "what concept that explains what is going on catches the latent pattern in this data?" and most important: "what are the participants main concern and how are they continually trying to resolve it?" Theoretical memos, in the shapes of text, diagrams, and figures, were written, typed, or drawn in the comparative process as soon as open coding started. This paper was sorted and written up from more than 4000 words and many dozens of pages of typed and handwritten memos. "Memos are the theorizing write-up of ideas about substantive codes and their theoretically coded relationships as they emerge during coding, collecting and analyzing data, and during memoing" [8]. Memoing is "the core stage of grounded theory methodology" [8], and should be done at any time and place in order to capture creative ideas. The analytic procedures were done with experience from earlier GT studies [13,14].

Discovery of Grounded Theory from 1967 [6] is the most quoted reference for any single method analyzing qualitative data according to Google scholar search (7884 citations August 2007). GT has the inductive approach to generate hypotheses explaining how participants in a studied substantive area resolve their main concern. Thus, GT conceptualizes "what is going on" in the field of study by the "constant comparative method", another name for GT [6]. This indicates a constant comparison of data during an iterative research process, which involves open coding, memoing, theoretical sampling (data collection based on hypotheses from the ongoing analysis), selective coding (recoding data based on concepts from the ongoing analysis), sorting and writing up (sorting memos in piles based on concepts in the theory and then writing up the sorted piles into a paper or book). GT analysis aims at conceptual theories abstract of time, place and people and differs from most studies using qualitative data by presenting explanatory concepts instead of descriptions. Many clinical research methods consider persons or patients as units of analysis, whereas in GT the unit of analysis is the incident [6] not the person(s) involved (incident = a distinct piece of action, or an episode, as in a story or play). The number of incidents being coded and compared typically amounts to several hundred in a GT study since every participant often reports many incidents. When comparing many incidents in a certain field, the emerging concepts and the relationship between them are in reality probability statements and therefore GT should not be considered a qualitative method but a general method that can use any type of data [9]. The results of GT are not reports of facts but an integrated set of conceptual hypotheses. Validity in its traditional sense is consequently not an issue in GT research, which instead should be judged by fit, relevance, workability, and modifiability [8]. Fit has to do with how close concepts fit with the incidents they are representing, and this is related to how thorough the constant comparison of incidents to concepts was done. A relevant study deals with the real concern of participants and captures attention. The theory works when it explains how the problem is being solved with much variation. A modifiable theory can be altered when new relevant data is compared to existing data. A GT is never right or wrong, it just has more or less fit, relevance, workability and modifiability, and readers of this article are asked to try its quality according to these principles.

## Results

In this study we analyzed student attitudes and "what was going on" in the medical ethics education based on survey data. The medical students argued for a proximity morality stance, or forming morality "on the job". This morality forming is ideally done in comprehensive ethics courses with tutored small groups. Forming physician morality requires "ethics grammar" provided by selected high quality lectures, and impulses from attitude exercises and vignette reflections in "ethics labs". Patient cases and clinical issues are thus discussed in interactive groups that help to deal with emotionally difficult clinical situations. To desist negative role modeling is another function of the ethics courses where reflected professionalism is developed for diverse medical students in a heterogeneous world.

### Proximity morality in medical school – How?

On the job morality forming in medical school is typically done in interactive discussion groups. These groups have a support network function where medical students are allowed professional role growth within a permissive context where ethical and value-laden issues are discussed and tried. The structure ideally consists of tutored groups that repeatedly work with case study approaches, discuss ethical principles, and continue during internship. Within a frame resembling the clinical setting students grow their own ethical attitudes and shape their individual physician morality. Group discussions provide good training for handling ethical difficulties since real world medical ethics consist of unique complex situations often involving several people. One goal of interactive ethics group discussions is to understand what appropriate physician behavior is.

*"ethics discussion forums should be based on tutored small groups to prevent people with strong views from dominating" *last term student.

*"(we need) group discussions with teachers making sure that everyone develops decent ethical values as physicians" *fifth term student

*"every section could end with ethical discussions related to the specific subject, psychiatry/internal medicine/surgery" *last term student

Forming physician morality also includes quality lectures on ethics, preferably by professional ethicists. These lectures provide students with a basic "ethics grammar" about ethical principles and concepts. This feeds the interactive group discussions and improves their quality concerning ethical issues.

*"Professional lecturers from the faculty of arts (are wanted)" *first term student

In the "ethics lab" students work with practical, sometimes challenging attitude exercises and vignette reflections. These stimulate critical thinking about current ethical problems in clinical training. It requires that participants position themselves ideologically, and for some attitude exercises also physically. Attitude exercises are often done in case studies.

*"A case is presented and different opinions (re the case) represented by four different corners. One can go to any corner and argue against the other corners and eventually change corners" *last term student.

### Proximity morality in medical school -Why?

Why would medical students want to form physician morality on the job? The deliberate forming of a physician morality is necessary for several reasons. A number of student responses dealt with arguments for ethics education in general and forming physician morality on the job in particular:

The professional identity of future physicians requires moral reflection.

*"An open discussion forum on difficult issues and professional identity conflicts would make us better physicians." *last term student

"*Small groups during clinical training – discussing the professional physician role and work issues *" (on suggested ethics education during later internship) last term student

Medical students are different. Some are ethically naive, or not interested in ethics, and others even described as socially "autistic". The importance of ethics education is obvious for these groups.

"*Only autistic people need ethics education*" last term student

Diversity. We live in a society with increasing diversity and multiple religious views.

*"What is it really like in our secularized country? How can we say something is right when we don't share the same values" *fifth term student

Processing difficulties. A group discussion format of ethics education helps in processing tough experiences from the clinical setting.

*"We underestimate the power of what we can do for each other during the education." *last term student

*"Small groups discussing everyday problems and ethical issues in the workplace" *(on suggested ethics education during later internship) first term student

Desisting negative role modeling. By defying ethics suppression and politically corrected ethics the influences of physicians/teachers as poor role models may be addressed and negative role modeling dealt with in the interactive groups. Some teachers and physicians were described as being "masters of opinion control" trying to neutralize discussions about ethically sensitive topics by putting the lid on discussions, and defending politically correct opinions.

*"I prefer a good (neutral) clinician instead of zealous, ideologically motivated people" *fifth term student.

*"Teachers gave too little space for own views – there was a correct key for the discussion" *last term student

In a statistical analysis of the survey presented elsewhere [4] we saw a significant relationship between a low interest in ethics and frequent experiences of poor role models and the absence of good ones in all three terms [OR 7.2 (CI: 1.2–43)]. For final-term students, there was a significant association between a high interest in ethics and experiences of good role models [OR 4 (CI: 1.4–11)] and a preference for discussions in small groups [OR 3.6 (CI: 1.4–9.5)].

## Discussion

"Personally I'm always ready to learn, although I do not always like being taught." 

W Churchill (1874 – 1965)

The quote illustrates the students' attitudes towards medical ethics education in this study. We propose that medical students by a proximity morality stance want ethics education on the job to help them in the learning process of becoming physicians. In this process they form their own physician morality rather than being taught ethics. This ideally takes place in comprehensive ethics courses where tutored groups openly discuss and reflect on difficult ethical topics and moral dilemmas. High quality lectures are interwoven to give an *ethics grammar*. These lectures provide default ethical principles nurturing group discussions together with attitude exercises and vignette reflections in *ethics labs *. These interactive discussion groups also have a support network function. Here students process ethical problems in an environment where physician morality is allowed to form and grow on the job. Hence, rather than being served ideologically stained opinions students prefer to reflect and discuss different ethical attitudes. As an example of their proximity morality one could say they want to bake their own moral cakes instead of being served ethical cookies [15].

In a British study of university students' expectations of teaching the students hoped for more interaction between students and teachers [16]. They also suggested that groups provide effective learning, and this view was most prominent among medical students. These findings resemble ours when it comes to preferences for teaching structures. In a Swedish study the authors suggested that interactive lecturing was a stimulant to a problem-based learning (PBL) program [17]. This is in line with our proposition of the need for good quality lectures to feed ethical discussions with *ethics grammar *and input from *ethics labs *. In a review of ethics teaching the authors were nihilistic about its effects and suggested that critical determinants of physician identity operate not within the formal curriculum but in a subtler, less officially recognized "hidden curriculum" [1]. Also, medical education could be seen as a form of moral training of which formal instruction in ethics constitutes only a small piece. In a study investigating the effect of ethics education on physician morality it was concluded that moral development and ethical confidence were unaffected by ethics education [18]. The goals of ethics education was conceptualized as having cognitive, behavior and attitudinal dimensions. Ethics was supposedly studied for its own sake contributing to "one's all around character". We agree with this author's conclusions, and our analysis suggests that instead of an emphasis on teaching, ethics and morality has to be learned on the job as discovered in a neonatal unit study of proximity ethics [19]. As a reference to oneself's morality Levinas talks about "the other" [20]. Similarly "the others" (fellow students and teachers/physicians) are necessary for understanding the suggested "on the job" morality development in our study.

The method for this study was classic grounded theory (GT) aiming at generating conceptual hypotheses of the resolution of a main concern and not describing a studied area. Yet, most studies citing GT fail to fulfill its aims [6-11]. In a Biomedcentral "grounded theory" search in 2006 the first author (HT) reviewed 35 consecutive articles. Some [21,22] presented a core variable – a fundamental part of GT, but most studies were descriptive and lacked explanatory integration [23]. In this study we did GT analysis of written open and multiple-choice survey responses. We did not theoretically sample data outside of the survey. Yet we conceptualized a tentative model, a preliminary core variable theory, of how medical students want their education in medical ethics. This model is, according to the GT paradigm, not right or wrong. It is just a set of probability statements from which hypotheses are generated by constantly comparing available data. When presenting this proximity morality model of ethics teaching to physician colleagues and ethics teachers the reactions have been mostly positive. The model makes sense and seems to fit with experience.

### Limitations

This paper suggests a proximity morality model showing how medical students want their ethics education in medical school, but does not take into account their teachers' views. Also, our study is limited by the qualitative data being only written comments [24] in an otherwise multiple-choice survey with a partial response rate. As for the low response rates, the centers with the highest response rates (83%) had the same attitude pattern as those with low response rates (13%) [4,5]. Thus the data seems generalisable enough to fit the requirements for an inductive study. The 11^th ^term students gave the largest quantitative input of qualitative data and thus had a comparatively larger impact on theory generation. Whether this was a limitation is questionable. In our view it gave us more valuable longitudinal data. To use interview data by theoretically sampling outside of the survey might improve the model. We tried to compensate for this by also sampling dichotomized multiple-choice survey data in accordance with the GT maxim "all is data". The survey data consisted of structured responses, which is not recommended in classic GT. This is another argument for expanding the theoretical model with interview data. For possible future application in medical schools we thus intend to refine and modify the model and develop it through interaction with medical students and teachers.

## Conclusion

In this study we present a tentative conceptual model of proximity morality guiding medical students to shape their own medical ethics education "on the job". We suggest that medical students rather than being taught ethics want to form their own physician morality. This is typically done in comprehensive ethics courses with tutored group discussions. Here high quality lectures supply "ethics grammar" and together with attitude exercises and vignette reflections in "ethics labs" nurture discussions on different ethical issues. This helps students to develop a professional physician identity, handling diversity of religious and existential worldviews, training students described as ethically naive, processing difficult clinical experiences, and desisting negative role modeling.

## Competing interests

The author(s) declare that they have no competing interests.

## Authors' contributions

RL and NL conceived the study. All authors helped in designing the survey questionnaire. RL and NL were responsible for coordinating the survey. HT did the grounded theory analysis and drafted the manuscript. NL, RL and KS gave input to the data analysis and manuscript. All authors read and approved the final manuscript.

## Pre-publication history

The pre-publication history for this paper can be accessed here:


